# Editorial: Genetic validation and its role in crop improvement

**DOI:** 10.3389/fgene.2022.1078246

**Published:** 2023-01-04

**Authors:** Ahmed Sallam, Ahmad M. Alqudah, P. Stephen Baenziger, Awais Rasheed

**Affiliations:** ^1^ Department of Genetics, Faculty of Agriculture, Assiut University, Assiut, Egypt; ^2^ Leibniz Institute of Plant Genetics and Crop Plant Research (IPK), Seeland, Germany; ^3^ Biological Science Program, Department of Biological and Environmental Sciences, College of Art and Science, Qatar University, Doha, Qatar; ^4^ Department of Agronomy and Horticulture, University of Nebraska-Lincoln, Lincoln, NE, United States; ^5^ Department of Plant Sciences, Quaid-i-Azam University, Islamabad, Pakistan

**Keywords:** genetic validation, QTLs, genetic improvment, molecular breeding approaches, crops

Gene discovery for economically important traits has remained a challenging Frontier in crop genomics and breeding. The recent advances in DNA sequencing technologies and genetic analysis approaches paved the way for discovering many genes and hotspot genomic regions controlling target traits. The detection of novel genomic regions or candidate genes is very useful for plant breeders and geneticists to improve crops, dissect the genetics of complex traits, and understand the biological mechanisms of genes underpinning traits of interest. Quantitative trait loci (QTL) mapping and genome-wide association studies (GWAS) dominated recent crop gene discovery research. These studies are becoming routine activities to discover the genetic basis of important phenotypes and result in underlying allelic variations, marker-traits associations, and frequency of favorable alleles in the target germplasm to help in understanding crop functional genomics (Rasheed and Xia 2019). However, the discovered loci need further validation before consideration to be used in breeding. In most GWAS cases, the outputs can be ambiguous due to problems of confounding population structure with low-frequency causal alleles leading to false-negative results and other unaccounted factors including low-accuracy genotype calls at some loci (Browning and Yu, 2009) and small population size (Finnoet al., 2014; Alqudah et al., 2020). Therefore, further validation is necessary, using cross-population approaches where candidate loci are either validated in bi-parental populations or independent germplasm collections (Finnoet al., 2014).

Genetic validation (QTL, genomic regions, candidate genes, gene expression, marker development, *etc.*) is one of the basic steps for marker-assisted and genomic selection for any breeding or genetic program to achieve its goals. Genetic validation examines whether the same QTL or gene tends to be significantly detected when the material is grown in other locations or years and whether its effect can still be significantly detected when tested in different genetic backgrounds (Sallam et al., 2016). Furthermore, validation of the polymorphic DNA markers in different populations is useful for further genetic diversity studies. The cost of sequencing and genotyping for genetic diversity studies could be an obstacle for some researchers. Providing validated and highly polymorphic markers could save time and effort in breeding programs.

The current Research Topic was planned to seek articles that aim to validate putative genetic results controlling target traits for the genetic improvement of crops. In total, 18 manuscripts were published in this Research Topic. The Research Topic covered many studies presenting proof of genetic validation which can be used for supporting future research studies.

Quan et al. conducted GWAS for salinity tolerance in wheat and identified five candidate genes including kinase family protein, E3 ubiquitin-protein ligase-like protein, and transmembrane protein. Tian et al. identified 41 loci in wheat underpinning such wheat flour properties and validated loci on chr3B and chr7B as important haplotypes. Likewise, Irshad et al., discovered nucleotide variations in gene encoding starch branching enzymes *TaSBEIII* and developed a KASP marker to identify the causal mutation. They also identified the allele frequencies of *TaSBEIII* in wheat collections from different countries. For gene validation, Ur Rehman et al. developed and validated KASP markers for drought tolerance related genes in wheat and validated their effect in different germplasm resources. Xu et al. demonstrated the effect of asymmetric somatic hybridization on synonymous codon usage bias in wheat. The validation approach was also used for disease resistant genetic region. Eltaher et al., identified and validated a high linkage disequilibrium region on chr1B, chr2A, and chr7B in wheat harboring stem rust resistance.

Maize lethal necrosis (MLN) is a viral disease with a devastating effect on maize production. Combined use of QTL and GWAS identified a major effect QTL, qMLN06_157, on chr6 and this QTL was proposed to be used in both marker-assisted forward breeding and marker-assisted backcrossing schemes to improve MLN resistance of breeding populations and key lines for eastern Africa (Murithi et al.). The maize (*Zea mays* L*.*) *ZmCNR13* gene, encoding a protein of *fw2.2-like* (*FWL*) family, has been demonstrated to be involved in cell division, expansion, and differentiation. In the present study, the genomic sequences of the *ZmCNR13* locus were re-sequenced in 224 inbred lines, 56 landraces and 30 teosintes, and the nucleotide polymorphism and selection signature were estimated (Zuo et al.). It was validated that natural variations of *ZmCNR13* might be involved in ear development and can be used in the genetic improvement of maize ear-related traits. Ahmed et al. evaluated maize inbred lines for pollen viability related traits under heat stress and identified five loci associated with these traits that had been validated and can be incorporated in maize breeding programs for improving heat stress tolerance.

Elattar et al. identified and validated seven major QTLs for soybean seed size and shape. Based on gene annotation analyses and RNA-Seq, they identified candidate genes which are potentially involved in seed-related traits.

The gelatinization of wheat flour is an important characteristic and useful in evaluating the eating and cooking quality of wheat. Mesocotyl is a crucial organ for pushing plants out of the soil, which plays a vital role in seedling emergence and establishment in direct-seeded rice. Eighteen QTLs for mesocotyl length were identified in rice, out of which 6 QTLs were validated in two mapping populations (Wang et al.). Further association analysis and gene expression studies confirmed the cross-population effect of two loci on chr1 and chr7 on mesocotyl length. Tomato is among the most valuable fruit crop and sugar and organic acids contribute to the overall flavor intensity of tomato. Metabolome and transcriptome studies in two tomato cultivars TM-1 and TM-38 identified that citric acid may play a more dominant role in the sugar/organic acid ratio of the tomato fruit (Li et al.). The contribution of both L-malic acid and citric acid to the fruit Brix was much greater than that of D-glucose and D-fructose. Genes involved in CHO and TCA metabolism, which have a significant correlation with the sugar/organic acid ratio were considered to be the contributing factors of fruit Brix.

Choudhury et al. used 36 SNP markers to identify the core set of 247 rice accessions from India’s east coast and validated the utility of using these SNP markers for the development of core collection and diversity studies. In another study, the genetic architecture of grain yield in wheat was revealed using a 50K SNP array, and 38 loci were identified (Li et al.). Khan et al. characterized the DECT1 gene in rice and the GUS and qRT-PCR analysis indicated that *DCET1* is specifically expressed in the anther till the developmental stage 9, consistent with the observed phenotype. The characterization of *DCET1* in callose regulation, pollen wall patterning, and tapetal programmed cell death (PCD) strengthens our knowledge for knowing the regulatory pathways involved in rice male reproductive development and has prospects in hybrid rice breeding. The phenomenal increase in the use of nitrogenous fertilizers coupled with poor nitrogen use efficiency is among the most important threats to the environment, economic, and social health. Sandhu et al. performed a meta-QTL analysis on 1,330 QTL from 29 studies published in the past 2 decades. A hot spot region associated with correlated traits on Chr 1, 4, and 8 and candidate genes associated with nitrate transporters, nitrogen content, and ammonium uptake on chromosomes 2, 4, 6, and 8 have been identified.

Iqbal et al. performed GWAS to identify the loci associated with single fiber quality in Asian cotton (*Gossypium arboreum*). They further identified 56 differentially expressed genes among the trait-associated SNPs to pinpoint the candidate genes.

Because, the functional gene validation is very important step to understand the mechanisms, Riaz et al. reviewed the recent breakthroughs in Clustered Regularly Interspaced Short Palindromic Repeats (CRISPR) and CRISPR-associated protein (Cas) mediated gene editing in cereal grasses and overviewed 25 different studies about it. The importance of CRISPR technology application in cultivated grasses improvement had been extensively explained.

In conclusion, a proof of genetic validation provides a very promising improvement in the production and productivity of crops for future research. Unfortunately, few research studies validated their genetic findings. The results of genetic validation in different crops (e.g. wheat, rice, maize, and cotton, *etc.*) presented in this Research Topic can be used for future and necessary genetic validation studies.
